# Diversity structure of culturable bacteria isolated from the Fildes Peninsula (King George Island, Antarctica): A phylogenetic analysis perspective

**DOI:** 10.1371/journal.pone.0179390

**Published:** 2017-06-20

**Authors:** Gerardo González-Rocha, Gabriel Muñoz-Cartes, Cristian B. Canales-Aguirre, Celia A. Lima, Mariana Domínguez-Yévenes, Helia Bello-Toledo, Cristián E. Hernández

**Affiliations:** 1 Laboratorio de Investigación en Agentes Antibacterianos. Departamento de Microbiología, Facultad de Ciencias Biológicas, Universidad de Concepción, Concepción, Chile; 2 Laboratorio de Ecología Evolutiva y Filoinformática. Departamento de Zoología, Facultad de Ciencias Naturales y Oceanográficas, Universidad de Concepción, Concepción, Chile; 3 Centro i~mar, Universidad de Los Lagos, Camino a Chinquihue 6 km, Puerto Montt, Chile; Massey University, NEW ZEALAND

## Abstract

It has been proposed that Antarctic environments select microorganisms with unique biochemical adaptations, based on the tenet ‘Everything is everywhere, but, the environment selects’ by Baas-Becking. However, this is a hypothesis that has not been extensively evaluated. This study evaluated the fundamental prediction contained in this hypothesis—in the sense that species are structured in the landscape according to their local habitats-, using as study model the phylogenetic diversity of the culturable bacteria of Fildes Peninsula (King George Island, Antarctica). Eighty bacterial strains isolated from 10 different locations in the area, were recovered. Based on phylogenetic analysis of 16S rRNA gene sequences, the isolates were grouped into twenty-six phylotypes distributed in three main clades, of which only six are exclusive to Antarctica. Results showed that phylotypes do not group significantly by habitat type; however, local habitat types had phylogenetic signal, which support the phylogenetic niche conservatism hypothesis and not a selective role of the environment like the Baas-Becking hypothesis suggests. We propose that, more than habitat selection resulting in new local adaptations and diversity, local historical colonization and species sorting (i.e. differences in speciation and extinction rates that arise by interaction of species level traits with the environment) play a fundamental role on the culturable bacterial diversity in Antarctica.

## Introduction

Antarctica hosts the last pristine ecosystems in the planet [1, 2], being exposed to low anthropogenic intervention [3, 4], and microbial communities remain unexplored. In fact, it has been proposed that its extreme environmental conditions, such as low temperatures, low precipitation, freeze-thaw cycles and high levels of UV radiation [5, 6] contribute to the selection of microorganisms having unique local biochemical adaptations [4, 7]. This idea, however, is based on the old microbiological tenet ‘Everything is everywhere, but, the environment selects’ by Lourens Gerhard Baas Becking [8]. While this idea has been widely used in environmental microbiology [9], it is a hypothesis that has not been extensively evaluated. This hypothesis adjusted to the mechanisms of community assemblage is based on classic niche theory, where species are structured in the landscape according to their requirements and its resources availability. This means that ‘environment selects’, therefore different habitats, independently of their geographical closeness and phylogenetic relationships of species within the community, will have a significantly different diversity due to different adaptation processes. This hypothesis implies that bacteria respond adaptively in short periods of time to environmental conditions which select them, independent of the particular history of lineage or ancestor-descendants relationships and habitat phylogenetic signal. However, the idea of Baas-Becking’s hypothesis of ‘environment selects’ opposes categorically the ‘phylogenetic niche conservatism’ hypothesis. Where, for this later, where related species are found to be ecologically similar and tends to retain similar ecological niches over evolutionary time scales [10–12]. This hypothesis implies that the use of particular habitat by bacteria lineages is a species-level attribute [13] (i.e. emergent trait, *sensu* Jablonski [13]), which is inherited from ancestors to descendants lineages. So the bacteria respond to the differential origination or persistence of species -together considered the emergent fitness of species within clades- owing to interaction with the environment or species selection -also termed species sorting- (for a review of the concept see Jablonski [13]). *Sensu* Jablonski [13], “species sorting shapes evolutionary patterns through differences in speciation and extinction rates (and their net outcome, often termed the emergent fitness of clades) that arise by interaction of intrinsic biological traits with the environment. From the ‘species sorting’ point of view, community assembly in local communities always goes to an endpoint that depends only on local environmental conditions [14], and the only role of dispersal is to occasionally replace species that go extinct [15] when true species-level characters interact with the environment to produce species sorting and trends [16, 17]. Consequently, stochasticity and dispersal limitation of community assemblages play an important role—like the proposed Hubbell’s Neutral Theory of Biodiversity [18]—in other words, habitats geographically closed do not differ significantly in their diversity, and species clades may be associated to their habitats by ‘phylogenetic niche conservatism’.

Antarctic microbial diversity has been studied mainly by means of culture independent techniques, which provide more information about microbial composition as compared with culture-dependent methods [19]. The latter are based on phenotypic characteristics, which compromise the resolution power for analyzing microbial composition of environmental samples. However, traditional procedures are still essential for isolating and to define the taxonomic and metabolic characteristic of pure strains [20], and also to understand how regional and local variability influence diversity spatial pattern in Antarctica [21].

An increasing number of studies have recently been carried out on bacterial communities in different geographical Antarctic regions [21–26]. However the bacterial diversity still accounts for only approximately 5% of the Antarctic species total record [21]. For example, the microbiological studies in Fildes Peninsula (King George Island) conducted in different types of environment (i.e. soils, cyanobacterial mats, sediments, lake systems and glaciers) [27, 28], suggests that bacterial diversity is heterogeneous and dependent on habitat type (i.e. Baas-Becking’s hypothesis). Nevertheless, these studies did not evaluate explicitly the prediction contained in this hypothesis based on formal statistical tests.

The aim of the present study was to evaluate the culturable bacterial diversity in different habitats in the Fildes Peninsula by means of 16S rRNA gene sequence analysis of cultured isolates. Also the phylogenetic relationships of the recovered isolates were determined by probabilistic approaches that includes data uncertainty, and based on this information we explicitly evaluate the fundamental prediction of Baas-Becking’s hypothesis: that the species diversity are structured in the landscape according to habitats that select them, and that these groups of species are not monophyletic since the local habitat selective pressures are independent from the lineages ancestor.

## Materials and methods

### Sampling method

**Fig 1:**
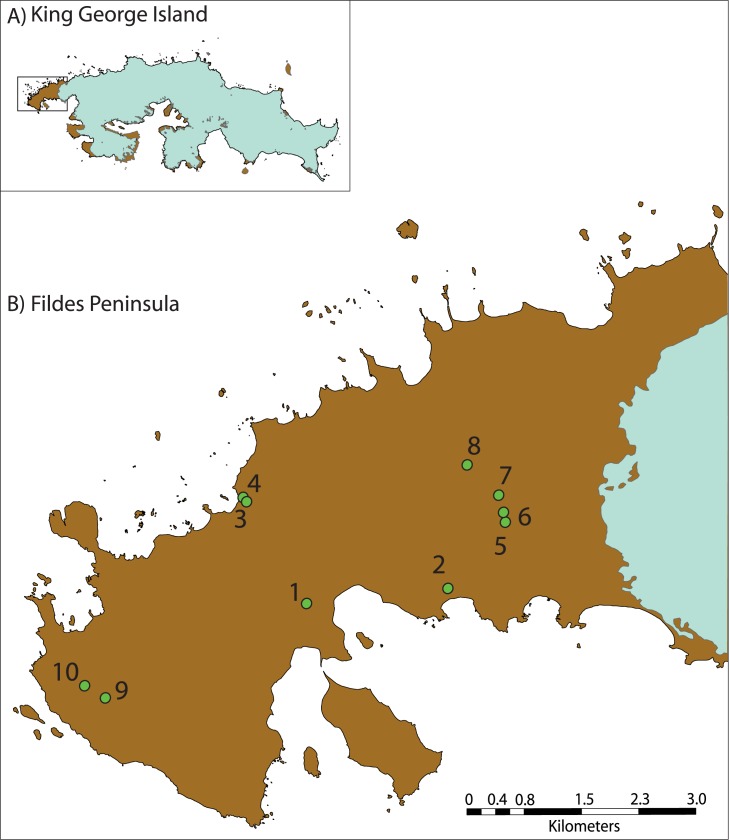
Location of sampling sites in Fildes peninsula, King George Island, Antarctica. Dots and numbers indicate the sampling sites: 1) Langer lagoon, 2) Russian fuel tanks, 3) Elephant seal beach, 4) Large valley, 5) North plateau, 6) High plateau north lagoon, 7) North plateau lagoon, 8) North plateau sun lagoon, 9) Jurasico lake, 10) Geografos lake.

Sampling was performed during the Antarctic Expedition ECA43 that took place in the Austral summer 2007, with the support of the Chilean Antarctic Institute (INACH). The samples included in this work were selected with the aim of including a variety of habitat-types from within the Fildes Peninsula. Samples of soil, mud, sediment, water, and ice were aseptically collected in the Fildes Peninsula, King George Island, using sterile 50 ml Falcon tubes (Table 1; Fig 1). The environmental temperature and that of the sample were registered in each sampling location. Equitable fractions of all samples were stored at 4°C and -20°C, and the former were processed within less than 24 hours at the laboratory in the Professor Julio Escudero Research Base, which belongs to the Chilean Antarctic Institute (abbreviated to INACH in Spanish). Permission to work in the study area and for microbiology samples was granted by the Chilean Antarctic Institute INACH (http://www.inach.cl/inach/), which considers the requirement to conform with all ethical considerations and the Protocol on Environmental Protection in the framework of The Antarctic Treaty, and related agreements or the Antarctic Treaty System, ATS (http://www.ats.aq/index_e.htm).

**Table 1:** Bacterial count of different samples from the Fildes Peninsula, King George Island, Antarctica.

Site	Sample ID	Location name	Lat / Long	Habitat	pH	T (°C)	CFU/g or CFU/ml	Strains
1	ANT-2	Langer lagoon	-62.204 / -58.967	Ice	8.1	0.1	3.7 x 10^2^	10
1	ANT-3	Langer lagoon	-62.204 / -58.967	Mud	8	4.1	2.2 x 10^6^	12
1	ANT-4	Langer lagoon	-62.204 / -58.967	Water	8	3.1	5.3 x 10^2^	8
1	ANT-5	Langer lagoon	-62.204 / -58.967	Soil under moss	7.6	2.8	2.2 x 10^5^	9
2	ANT-8	Russian fuel tanks	-62.194 / -58.938	Soil	ND	4.4	7.6 x 10^6^	5
3	ANT-31	Elephant seal beach	-62.197 / -58.993	Water	7.2	6.2	9.5 x 10^4^	5
4	ANT-32	Large valley	-62.197 / -58.993	Soil	ND	6	1.1 x 10^5^	1
5	ANT-33	North plateau	-62.184 / -58.934	Sediment	9	0	2.0 x 10^4^	3
6	ANT-34	High plateau north lagoon	-62.183 / -58.936	Water	9.5	2.3	2.0 x 10^2^	4
6	ANT-35	High plateau north lagoon	-62.183 / -58.936	Soil	7.26	3.5	7.5 x 10^3^	5
7	ANT-36	North plateau lagoon	-62.182 / -58.939	Water	9	2.2	2.0 x 10^2^	5
8	ANT-37	North plateau sun lagoon	-62.181 / -58.950	Water	9.18	0.7	5.3 x 10^2^	3
9	ANT-39	Jurasico lake	-62.224 / -58.998	Water	6.4	5.1	6.7 x 10^2^	5
10	ANT-40	Geógrafos lake	-62.224 / -59.004	Water	6.2	5.4	5.5 x 10^1^	5

ANT: Antarctica, Lat: Latitude, Long: Longitude, CFU: Colony-Forming Units, Strain: number of strains isolated, ND: No data

### Culture, count and isolation of aerobic heterotrophic bacteria

Depending on the type of substrate, 1 g or 1 ml sample was thoroughly suspended in 9 ml of sterile distilled water using a Vortex mixer. Serial dilutions ranging from 10^−1^ up to 10^−4^ were performed, and 100 μl of the supernatant of each dilution were spread on plates containing the oligotrophic medium agar R_2_A (BD Difco^TM^) supplemented with cycloheximide (50 mg/L) to inhibit the growth of fungi [29], followed by incubation at 20°C in aerobic conditions for 5–10 days until the number of colony-forming units (UFC) remained unchanged. Only those plates containing 30–300 colonies were used to calculate total bacterial counts. Morphotypes that differed macroscopically (i.e. morphology, size, pigmentation, etc.) were subcultured, isolated, and purified using the same culture medium. Pure bacterial isolates were stored at -80°C in 50% v/v glycerol in 0.5x R_2_A broth.

### DNA extraction, amplification and sequencing of 16S rRNA gene

Total DNA was obtained from bacterial colonies resuspended in 200 μl of 5% Chelex and 2.5 μl of proteinase K (20 mg/ml) in DNAse free water, followed by incubation at 56°C for 45 min and boiling for 8 min. The samples were then centrifuged at 16000 g for 1 min in a microcentrifuge (Eppendorf). The supernatant was stored at 4°C and used as template in PCR to amplify the 16S rRNA gene (1500 bp) using primers P0 (5’-GAGAGTTTGATCCTGGCTCAG) and P6 (5’-CTACGGCTACCTTGTTACGA) [30]. We used these universal primers as they have been employed for the successful amplification of the 16S gene in a wide range of bacterial species, and are thus considered universal [31–33]. Also, in next generation sequencing protocols, this same marker (16S rRNA gene) is widely used to assess microbial diversity [34].The PCR mixture consisted of 2.5 μl of 10x PCR buffer, 1.25 μl of 50 mM MgCl2, 0.2 μl of 25 mM dNTPs, 0.4 μl of each primer (P0, P6, 25 pmol/ μl), 0.25 μl of *Taq* DNA polymerase (5 U/μl) and 1 μl of template DNA in a final volume of 25 μl. The amplification was carried out in an Eppendorf thermal cycler (Mastercycler Personal 5332) under the following conditions: initial denaturation at 95°C for 2 min followed by 30 cycles of denaturation at 95°C for 90 sec, annealing at 52°C for 60 sec and extension at 72°C for 90 sec; and a final extension at 72°C for 15 min. The PCR products were subjected to electrophoresis in an 1.5% agarose gel at 100 v for 45 min, which was then stained with ethidium bromide and visualized on a transilluminator under UV light. PCR products were purified with ExoSAP-IT methods and sequenced using primer P0 in Macrogen Inc. (USA) by an ABI 3730XL sequencer (Applied BioSystems). All sequences were deposited in GenBank under the following accession numbers: KC912567–KC962646.

### BLAST-based identification of strains

The resulting 16S rRNA gene sequences were compared to those available in the GenBank database using BLAST (Blast Local Alignment Search Tool) [35], in order to identify the closest relatives of each isolated strain. The dataset from BLAST used to compare was downloaded from GenBank in January 23, 2013. Additional information about the resulting highest scoring hits (≥99% sequence similarity were considered different phylotypes) was obtained, such as isolation source and country where samples were obtained, in order to compare these hits to our samples and assess any concordance in biome or habitat type (S1 Table).

### Molecular phylogenetic analysis

In order to avoid obtaining spurious outcomes resulting from the lost phylogenetic information due to substitution saturation, we tested whether the sequences used were useful for the phylogenetic analysis thought the Xia’s test [36] implemented in DAMBE v5.1.5 [37]. Xia’s test is an entropy-based index that estimates a substitution saturation index (Iss) and compares the Iss to a critical substitution saturation index (Iss.c) via a randomization process with 95% confidence intervals [36, 37]. We used DNA aligned sequence data of the 16S rRNA fragment gene of 80 environmental strains isolates in this study (S1 Table), and 46 sequences of several bacterial genera obtained from GenBank (www.ncbi.nlm.nih.gov/). The sequence selection criteria were: (1) that these sequences present a similar length (size in bp), and overlapped with sequences obtained in this study, thus we optimized the number of homologue characters used in the comparison; (2) include the greatest number possible of representative members of each morphological clade, so as to optimize the species allocation and strengthen the phylogenetic analysis results; and (3) that the sequences, at least, were published in a scientific journal (see S2 Table). The comparative analysis was conducted in order to assess the biodiversity of the environmental samples isolated and to reconstruct phylogenetic relationships of strains of Antarctic bacteria. The phylogenetic analysis described was conducted using 126 sequences plus an outgroup (i.e., *Aquifex pyrophilus*; GenBank accession M83548) (See S1 and S2 Tables). The purpose of this outgroup was to root the phylogenetic tree, and we choose this sequence because it has been used as outgroup in several other studies (from Antarctic or other places) that include environmental bacteria as the focus of research [e.g. 38, 39–43]. The alignment was performed using SEQUENCHER v5.0 (Gene Codes Corporation) and trimmed-end by visual observation.

The phylogenetic analysis was conducted applying a general likelihood-based mixed model (MM) of gene sequence evolution as described by Pagel and Meade [44, 45], that accommodates cases in which different sites in the alignment evolved in qualitatively distinct ways, but does not require prior knowledge of these patterns or partitioning of the data. The Reversible-Jump Markov Chain Monte Carlo (RJMCMC) procedure [46] was used with the objective of finding the best MM that summarizes the sequence evolution, using the BAYESPHYLOGENIES 1.1 software. This approach enables researchers to explore the variety of possible models and parameters, converging towards the model that best fits the data in the posterior tree sample. One RJMCMC analysis was run using 29,000,000 generations of phylogenetic trees, sampling every 10,000th tree to assure that successive samples were independent. From this sample of trees, the 25% of the sample was removed to avoid including trees sampled before the convergence of the Markov Chain, and we obtained a final sample of 2,176 trees. These trees were used to obtain the phylogenetic consensus tree with the posterior probability (pp) of the nodes.

### Testing Baas-Becking’s and phylogenetic niche conservatism hypotheses

To assess if different habitats (i.e. ice, mud, sediment, soil and water) have a significantly different diversity (i.e. different phylotype depending of the habitat) by different adaptations processes as predicted by Baas-Becking’s hypothesis of ‘environment selects’ the statistical significance of the differences in bacterial communities among habitats (i.e. ice, mud, sediment, soil and water) was evaluated by a one-way Analysis Of Similarity ANOSIM based on the Jaccard distance Index [47]. The ANOSIM test is a non-parametric statistical test widely used in the field of ecology, and has some similarity to an ANOVA-like hypothesis test, however, it is used to evaluate a dissimilarity matrix rather than raw data [47]. For this statistical analysis, the replicates for the different habitat types were the samples, which comprised the compared groups. Specifically, ANOSIM, analog to an Analysis Of Variance (ANOVA), compares the distances between groups (i.e. habitats) with the distances within groups, using the statistical *rb* or mean rank of all distances between groups, and *rw* or mean rank of all distances within groups for calculating the value of R, which allows us to evaluate dissimilarity between groups (i.e. habitats), where: Positive values of R (0 < R ≤ 1) indicate differences between groups, whose significance was estimated by means of 50,000 permutations of the observed values. If the analysis does not show significant differences (p> 0.05), it implies that there are no differences between habitat types in terms of diversity (i.e. different phylotype diversity not depending of the habitat), and consequently there would be no support for Baas-Becking’s hypothesis. This analysis was conducted using the software PAST 3.0 [48].

Finally, to evaluate the extent to which the phylotypes of the phylogenetic tree was adjusted to the ‘phylogenetic niche conservatism’ hypothesis of analyzed habitats, the phylogenetic signal of the habitat type was evaluated using the software BaTS [49] by means of the Association Index (AI) and using a Bayesian framework in the sample of trees obtained from the phylogenetic analysis. This index measures the phylogenetic autocorrelation in discrete characters (e.g. habitat type), i.e. it statistically evidences if phylogenetically close species (same clades) tend to resemble themselves more than it could be expected by chance alone [50], given their discrete characteristics (in our case habitat type). In this analysis the null hypothesis is that features (habitat) are randomly distributed in phylogeny, or the lack of association between the ancestor-descendants history of the lineages (structure of monophyletic clades) and the analyzed variable [51], which in this case is the habitat. We evaluated the statistical significance of the distribution values of AI observed in the phylogenetic tree sample comparing it with the null probability distribution of AI values obtained from a total of 20,000 randomizations. If the analysis shows significant differences (p< 0.05), it implies that the habitats are not randomly distributed in the phylogeny, or given the habitat type, the clades tend to resemble themselves more than expected by chance alone, which would consequently support the ‘phylogenetic niche conservatism’ hypothesis.

## Results

### Viable bacterial counts

In general, bacterial colonies were visible after 3–4 days at 20°C, and for all samples, R_2_A was found to be suitable growth medium at this temperature. Most colonies were pigmented and displayed regular edges. Variations in color and morphology were used as a criterion for selecting pure isolates. A total of 80 isolates were selected from 10 sampling sites listed in Table 1. The bacterial counts in all types of habitats varied between 5.5 x 10^1^ (Water sample from Geografos Lake) and 7.6 x 10^6^cfu/g (Soil sample from Russian Fuel tank). On the other hand, the substrate water, showed the lowest bacterial count, ranging between 5.5 x 10^1^ and 9.5 x 10^4^cfu/ml. The number of bacteria recovered appears to be dependent upon the type of substrate from which environmental samples were obtained (Table 1). The number of morphotypes retrieved per type of habitat ranged from 1 to12 (Table 1).

### BLAST-based identification of strains

**Fig 2:**
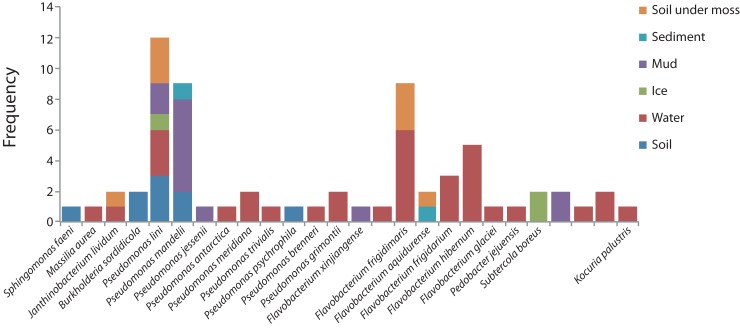
Frequency of phylotypes isolated by habitat. Differentiation based on membrane permeability showed that 21 of 26 phylotypes were comprised by Gram-negative strains (i.e. phyla Proteobacteria and Bacteroidetes), and 5 of 26 by Gram-positive strains (i.e. all to phylum Actinobacteria) (S1 Table). Strains of *Pseudomonas* (n = 34), *Flavobacterium* (n = 22), and *Arthrobacter* (n = 3) were frequently isolated and identified at genus level, and belong to the phyla Proteobacteria, Bacteroidetes, and Actinobacteria, respectively (S1 Table). We compared the isolation source of strains from this study with strains representing their nearest phylogenetic relative (NPR) in the NCBI database. Forty two of 67 NPR have a non-Antarctic isolation source (S1 Table). However, 25 of 67 NPR were isolated from Antarctic or similar environments, specifically from habitats like mats, glaciers, seawater, freshwater, and marine sediments (S1 Table). Finally, identification of the different phylotypes showed that only 5 of them were recovered from two or more habitats (i.e. *Janthinobacterium lividum*, *Pseudomonas lini*, *Pseudomonas mandelii*, *Flavobacterium frigidimaris*, *Flavobacterium aquidurense*) (Fig 2). The most frequently encountered phylotypes were *Pseudomonas lini*, and *Pseudomonas mandelii*, which were found in 5, and 3 habitats respectively, from a total of 6 different analyzed habitats (Fig 2).

Comparative sequence analyses between representatives of each phylotype and bacterial sequences available in the GenBank database showed that similarity percentage ranged from 93 to 100% (S1 Table). According to similarity criterion ≥ 99% (1% divergence) for the 16S rRNA gene sequence, 67 of 80 strains isolated from 10 sampling sites were classified as 26 different phylotypes (S1 Table, Fig 2).

### Phylogenetic analyses

The result of Xia’s test suggests that 16S rRNA gene sequences presented low saturation, as the critical index of substitution saturation value (Iss.c = 0.733) were significantly higher than the observed index of substitution saturation values (Iss = 0.267; *p* < 0.0001), therefore, the sequences were deemed suitable for performing phylogenetic analyses.

**Fig 3:**
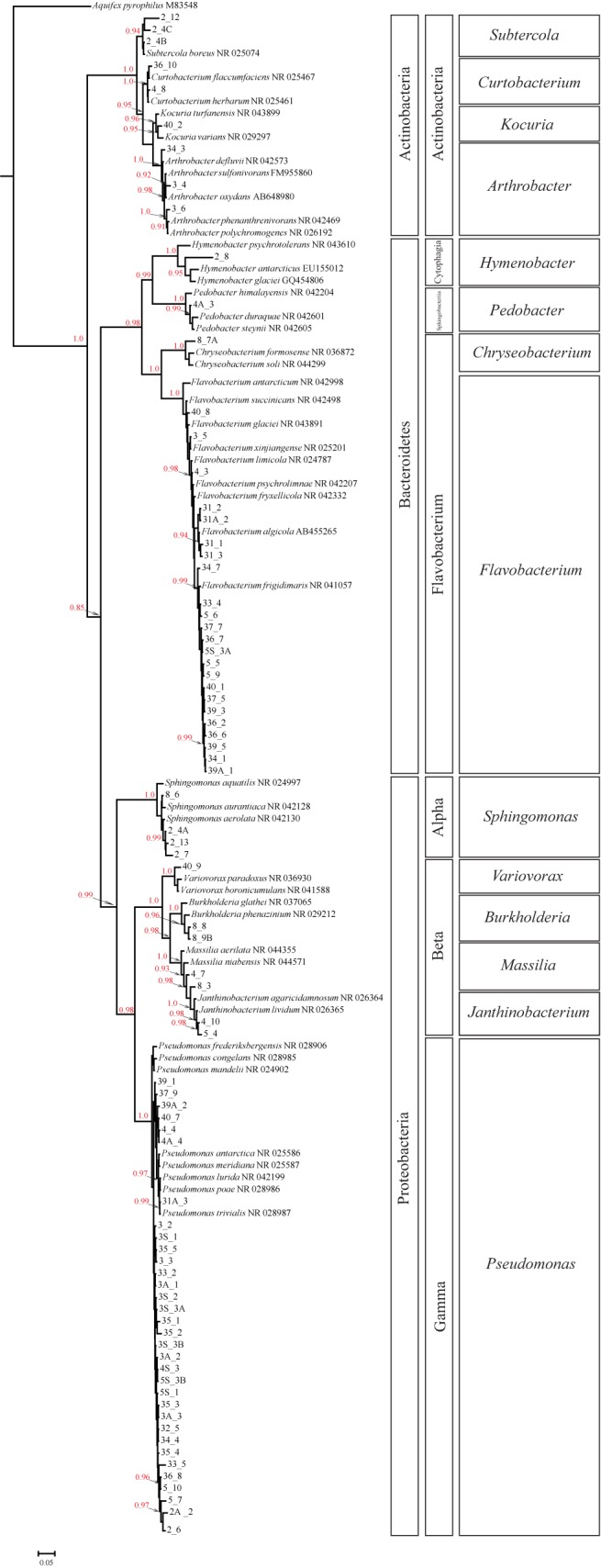
Bayesian consensus tree based on RJMCMC approach of the 16S rRNA gene sequences of the environmental Antarctic strains isolated and closely related species obtained from GenBank. The codes indicate the environmental Antarctic strains isolated (see S1 Table). The GenBank accession number follows specific names of the closely related species found in the database. The red numbers at tree nodes are posterior probabilities values (only >0.9 posterior probability) obtained from 2176 samples of phylogenetic trees. Scale bar represents 5% estimated substitutions.

*Pseudomonas* and *Flavobacterium* were the most representated genera in the culturable bacteria isolated from Antarctic´s habitats, 41% (33 of 80) and 29% (23 of 80), respectively. Within the *Pseudomonas* and *Flavobacterium* clades, strains showed subtle differences among culturable and GenBank’s species. Nonetheless, only four clades showed high posterior probabilities for each genera. The BMCMC approach showed high posterior probabilities on the nodes (Fig 3). The tree root displayed high posterior probability (*p*-value = 1.0) suggesting a monophyletic status of the culturable bacteria isolated from Antarctic´s habitats (excluding outgroup). Monophyletic phyla based on posterior probability were Actinobacteria (*p*-value = 1.0), Bacteroidetes (*p*-value = 0.85), and Proteobacteria (*p*-value = 0.99), as well as almost all genera (Fig 3). In addition, the Proteobacteria Classes (Alpha, Beta and Gamma) showed posterior probabilities of 1.

### Testing Baas-Becking’s and phylogenetic niche conservatism hypotheses

The Analysis Of Similarity ANOSIM does not show significant differences of bacterial communities’ composition among habitats (i.e. ice, mud, sediment, soil and water) (R = 0.1881, *p* = 0.1232), which supports a lack of local habitat community structure of the Antarctic culturable bacteria in the Fildes Peninsula.On the other hand, the analysis of phylogenetic signal, based on the Index of Association (AI), shows that different habitat types are occupied by phylogenetically close phylotypes, or that the phylotypes inhabiting a particular habitat display a higher degree of relatedness than expected by chance alone (Observed mean AI = 2.932, lower 95% CI = 2.243; upper 95% CI = 3.654; Null mean AI = 5.489, lower 95% CI = 4.861, upper 95% CI = 6.113; Significance *p*-value < 0.0001). This means the phylotypes that inhabit some habitat depend from the habitat of the lineages ancestor.

## Discussion

We evaluated the Baas-Becking’s and phylogenetic niche conservatism hypotheses to answer if the culturable bacterial diversity in different habitats from Fildes Peninsula, Antarctica is structured in the landscape according to habitats that select them, or if these groups of species are not monophyletic since the local habitat selective pressures are independent from the ancestor lineages. According to our results, culturable phylotypes do not group significantly by habitat type, which does not support the Baas-Becking hypothesis. Nonetheless local habitat types showed phylogenetic signal between the culturable bacteria, which support the phylogenetic niche conservatism hypothesis.

### Culturable isolated bacteria

As described by Peeters and Willems [3], an oligotrophic media such as R_2_A [52] is one of the most favorable culture media to isolate Antarctic bacteria [3]. Using this growth medium, we isolated 80 strains, from which 26 represent unique phylotypes based on ≥ 99% similarity, and most of the isolates produced colonies that exhibited an intense color due to pigment production. Coloration may be a characteristic that provides crucial protection against oxidative stress caused by the high levels of UVB radiation in Antarctica [53, 54].

Twenty-six phylotypes based on ≥ 99% similarity to the nearest phylogenetic relative in GenBank allowed a likely species assignation. However, although a>97% similarity in the 16S rRNA gene sequence has been proposed as a threshold for species delineation, as it generally correlates to 70% DNA-DNA whole genome hybridization [55], some authors have proposed a stricter threshold of 99% similarity [34]. Only five isolates yielded a closest hit with a 16S rRNA sequence below 97%: isolate 2_8 (93%), isolate 2_7 (95%) and isolate 8_7A (96%) (S1 Table), strongly indicative of putative new species and even a member of an uncharacterized novel genus. These isolates are available in our strain collection for further phenotypic and genetic analyses. The majority of isolates (66) yielded hit with 99% sequence similarity, indicating kinship at species level, although only 2 isolates yielded 100% identity matches. These results support the notion that using culture dependent techniques allows the isolation of microorganisms not previously described [33]. Nonetheless, to be able to enlighten a real taxonomic association of these isolates it is necessary to consider both phenotypic data (e.g. biochemical tests, and fatty acids composition), and genotypic information (e.g. DNA fingerprint data) [56].

The most representative group found in this study was the Phylum Proteobacteria (60%), in agreement with what was found by Yergeau et al.[6] in a study of bacterial communities from Antarctica using DGGE. Based on pyrosequencing Teixeira et al.[2] reported that Firmicutes was the dominant phylum in almost all but one sample where Proteobacteria was the most abundant phylum. These differences could be related to the lower sensibility of using culture-based techniques to assess diversity compared with pyrosequencing, in particular when rare taxa are to be found [2]. Similarly, Wang et al. [57], who has recently surveyed the diversity and structure of the microbiota associated with soils in the Fildes Peninsula by culture-independent pyrosequencing, identified over 15,000 operational taxonomic units (i.e. Proteobacteria, Actinobacteria, Acidobacteria and Verrucomicrobia). Taking into account the limitations associated with the identification of isolated culturable bacteria compared with non-culturable -methods (i.e. NGS technology), we also found Proteobacteria and Actinobacteria (S1 Table). However, interestingly Wang et al. [57] did not identify any members of the Bacteroidetes phylum, which was represented by eight phylotypes belonging to four genera in our survey and, as a whole, accounted for about 30% of the identified phylotypes. However, Wang et al. [57] only sampled surface soils, while most Bacteroidetes in our study were isolated from water, as well as from mud and sediment or from samples collected under moss, with only one surface soil sample positive for Bacteroidetes.

### Culturable isolated bacteria vs NGS methods

As we stated before, next generation sequencing allows direct sequencing and identification of bacterial taxonomic units and is much more powerful in identifying total diversity, and we are aware that culture methods will unavoidably narrow the number of species analyzed. However, isolation by culture allows for a full description and characterization of strains including the potential assignation of new species. However, our results based on culturable methods, are also able to unveil hidden biodiversity in the Antarctic environment, as represented by the two main groups i.e. *Flavobacterium* and *Pseudomonas* genus (Fig 3). This reinforces confidence in culturable methods for this kind of studies, as culturable methods allow not only classifying bacteria, but also characterizing the physiology and functionality of the colonies obtained. In addition, isolation by culture also allows for the analysis of the complete 16S rRNA gene (~1500 pb), providing enough resolution power to answer the big question presented in this study, while NGS approaches generally provide shorter reads. Thus, taking this into account it may not be compulsory to use novel NGS technologies to discover new biodiversity. Furthermore, similar results from recently NGS surveys, based on 16S rRNA, about diversity groups have been found in the Antarctica. For instance, Tytgat et al. [58] found that the majority of genera retrieved by pyrosequencing were not found by culture, but interesting 25% of those identified by culturing were not retrieved by pyrosequencing, illustrating that each technique has intrinsic advantages and limitations. Similarly to our work members of Proteobacteria, Actinobacteria, Bacteroidetes were identified by culture-dependent methodology by Tytgat et al. [58], and corresponded to the most abundant recovered phyla by pyrosequencing together with Firmicutes. Nevertheless, Firmicutes were also recovered by culture, but were not present among our isolates, which is may be due to the specific matrix from which samples were taken (mats) and the differing methodology employed. Similarly, Luria et al. [59] also characterized the microbial community diversity and composition along the Antarctic coast; they found that bacterial richness showed no differences between sampling sites located inshore versus offshore, but did vary with depth and season, reflecting perhaps differing physical parameters. On average 234 OTUs were recovered from each site and the most abundant sequences retrieved belonged to the Proteobacteria group, with Flavobacteria also commonly identified [59]. Likewise, Lopatina et al. [60] analyzed the composition of bacterial communities on surface snow samples from Antarctica. Although different snow samples yielded somewhat different results, in all four sites Flavobacteriia was abundant. Likewise in our work members of this genus were frequently isolated, even though our sampling sites are located on the opposite side of the continent. In their work Flavobacteriia together with Proteobacteria (Alpha, Beta, and Gamma) account for between 70% and 90% of abundance in three out of the four sampled sites; these two groups also account for most isolates in our work [60].

Finally, based on non-culturable studies (i.e. NGS approach), distance has not been a deterministic process in the bacterial community composition in high ice covered habitats, in Arctic and Antarctic environments[61, 62]. However, some physical-chemical parameters of habitat sampled such as salinity, temperature and solar radiation showed a strong relationship to bacterial community composition.

### Phylogenetic relationship and source of isolates from GenBank

Our phylogenetic tree shows 80 bacterial isolates clustering in a topology of three main clades (Actinobacteria, Bacteroidetes and Proteobacteria). These clades are quite distinct due to the large amount of changes occurred along the lineages, supported by a high *a posteriori* probability value in the nodes (Fig 3). The relationship within each clade was very close among the study strains and members of genetically related genera (Fig 3) due to the high level of conservation of the 16S rRNA gene sequence, which may limit the resolution power of this molecule at the level of closely related species [20]. Nonetheless, the phylogenetic relationship of the three phyla found in our analysis based on culturable bacteria isolated from the Fildes Peninsula agreed with that found by Hug et al. [63] based on genomic data; supporting that bacterial deep phylogenies could be accurately summarized with few representatives of each phylum. In the same way, at Class level, relationships among representatives of Proteobacteria matched with the genome survey carried out by Hug et al. [63], unlike the relationship among Classes of Bacteroidetes. For the latter, we found that Flavobacterium is the sister group of the clade that includes Cytophagia and Sphingobacteria, however, Hug et al. [63] showed that Cytophagia is the sister group of the other two aforementioned Classes.

Based on isolation source of the nearest phylogenetic relative (NPR) from GenBank that matched with the phylotypes isolated here, we found that 37.3% of the NPR were isolated from the Antarctica. For *Pseudomonas* and *Flavobacterium*, the main genera isolated here, the Antarctica isolated source was different. According to the phylogenetic analysis some identical lineages within the clade formed by *Pseudomonas* were found to inhabit lake and terrestrial systems geographically far apart (S1 Table; Fig 2), possibly because members of this genus exhibit a wide metabolic versatility, a trait that allows them to successfully occupy distinct habitats [64]. However, three isolated strains matched with a NPR isolated from Antarctica (S1 Table). On the other hand, in the *Flavobacterium* genus the majority of the isolation sources of the NPR were associated to water habitats from Antarctica [65–70] (S1 Table). For both, *Pseudomonas* and *Flavobacterium*, while GenBank phylotypes and strains showed subtle differences, even non-significant, our results suggest that some of these new isolates might be undescribed types of *Pseudomonas* and/or *Flavobacterium*.

Other less represented phylotypes found here have also been found in habitats from Antarctica. For example, the members of the genus *Chryseobacterium* (class Flavobacteria) have been originally isolated from soil in King George Island [71], the genera *Hymenobacter* (class Cytophagia) and *Pedobacter* (class Sphingobacteriia) have been previously detected in Antarctic habitats [72–74]. Finally, for Actinobacteria, all phylotypes matched a NPR isolated from a similar environment to Antarctica. Specifically, 2-4B and 2-4C that matched with the species *Subtercola boreus* isolated from the groundwater of a shallow aquifer located under a glacial gravel ridge in Southern Finland [75]. Lastly, none of those phylotype related to Alphaproteobacteria, Betaproteobacteria and Actinobacteria showed an exclusive Antarctic distribution (S1 Table). Only six phylotypes within the phylum Bacteroidetes, and two phylotypes within the Class Gammaproteobacteria, have an Antarctic origin at present (S1 Table). Conclusively, at present it is not possible to definitely state that certain bacterial taxa are exclusive to Antarctica, since knowledge about global bacterial diversity is still limited [21, 76].

### Testing Baas-Becking’s and phylogenetic niche conservatism hypotheses

The results of structure per habitat (i.e. ANOSIM) show that phylotypes do not group significantly according to habitat type, but the phylogenetic signal (i.e. AI) shows that the habitat occupied by bacterial isolates was dependent on their phylogenetic relationships, or their ancestors. In other words, the ‘phylogenetic niche conservatism’ hypothesis is supported for culturable Antarctic bacteria in the Fildes Peninsula, and these bacteria tend to retain similar ecological niches over evolutionary time scales. Similarly, Moroenyane et al. [77] found that distantly related OTUs do tend to co-occur given their phylogenetic signal, indicating that deterministic processes could play a major role in assembling the bacterial community. As a consequence, our results do not support the old microbiology tenet “*Everything is everywhere*, *but*, *the environment selects*” by Baas-Becking [8], i.e. the environment does not select bacterial communities on a local scale. Our outcomes are in disagreement with what was recently found by Fondi et al. [78], which supported the Baas-Becking hypothesis based on 339 metagenome projects, suggesting that it also applies to genes [78]. However, among the metagenomes used by Fondi et al. [78], only three corresponded to sampled habitats from Antarctica. Nonetheless, our results based on culturable bacteria would support Hubbell’s Neutral Theory of Biodiversity [18] in Antarctic environments, where stochasticity and dispersal limitation of community assemblages plays a crucial role on a historical scale. In other words, diversity of geographically closed habitats does not differ significantly, but clades of species are associated to habitats, on which depend the probability of local stochastic colonization and extinction. In addition, although bacteria specific to each habitat were found, in general the geographic distribution of phylogenetic lineages did not seem being dependent on the type of habitat. It was even observed in two cases the presence of the same lineage in habitats quite distant from each other, a phenomenon that could probably be explained by a high microbial dispersion in the Peninsula or by the metabolic versatility of these lineages. This study is the first report on the phylogenetic relationship among culturable bacteria found in different habitat types that include water, soil, sediment and ice, in the Fildes Peninsula. Thus, we propose that, more than habitat selection resulting in new local adaptations and diversity, local historical colonization and species sorting play a fundamental role on the culturable bacterial diversity in Antarctica.

## Supporting information

S1 Table Identification of bacterial strains isolated from the Fildes Peninsula, Antarctica. (XLSX)

S2 Table List of sequences used to build the phylogenetic relationships. Names in bold correspond to phylotypes.
(XLSX)
